# 

**Published:** 1891-07

**Authors:** 


*American Veterinary College,*

*New York.*

JULY, 1891.

CONTENTS:

PAGE

ORIGINAL ARTICLES:

The Biology of the Cattle Tick. By Cooper Curtice, Vet. .... 313

Does Menière's Disease Occur in Horses? By George Fleming, C. B., LL.D., F. R. C. V. S. .... 319

Remedies. By Dr. Herbert Neher .... 325

Traumatic Hernia. By J. Wallace Marsh, D. V. S. .... 326

Age of the Horse, Ox, Dog, and Other Domesticated Animals. By R. S. Huidekoper, M. D., Vet. .... 327

Locomotor Ataxia in the Horse. By Louis Magnin, Vet. .... 333

Dicephalus-Bicollis Monstrosity. By Fred. H. P. Edwards .... 336

Recent Patents. .... 337

Tuberculosis in a Boar. By M. S. Mayo, M. S., D. V. S. .... 345

Hyposulphite of Soda. By J. W. Marsh, D. V. S. .... 348

EDITORIAL:

Dr. C. B. Michener .... 344

United States Veterinary Medical Association .... 344

New York State Veterinary Medical Society .... 345

Tuberculosis .... 345

SELECTIONS FROM FOREIGN JOURNALS:
German Review .... 346

SOCIETY PROCEEDINGS:
Connecticut Veterinary Medical Association .... 349
Massachusetts Veterinary Association .... 351

VETERINARY COLLEGE NOTES:
Lahore Veterinary College .... 354
University of Pennsylvania, Veterinary Department: Commencement 357
Chicago Veterinary College .... 357
Harvard University, Department of Veterinary Medicine .... 357
Ontario Veterinary College .... 358
Alumni Association of the American Veterinary College .... 358

COMMUNICATIONS:
Osteo Porosis. By V. G. Hunt, V. S. .... 359

REVIEW DEPARTMENT:
17th Annual Report .... 361
Books and Pamphlets Received .... 361

